# Association of Body Mass Index with Early Inflammatory and Coagulation Biomarker Patterns and Intensive Care Unit Mortality in Critically Ill Patients with COVID-19: A Retrospective Cohort Study

**DOI:** 10.3390/jcm15207799

**Published:** 2026-10-09

**Authors:** Mikołaj Korabiewski, Izabela Płońska-Korabiewska, Sławomir Lech Czaban, Jerzy Robert Ładny, Mateusz Bartoszewicz

**Affiliations:** 1 Department of Anesthesiology and Intensive Care, Medical University of Bialystok, 15-089 Bialystok, Poland; mikolaj.korabiewski@gmail.com (M.K.); slawomir.czaban@umb.edu.pl (S.L.C.); 2 Medical University of Bialystok, 15-089 Bialystok, Poland; plonska.izabela@gmail.com; 3 Department of Emergency Medicine, Medical University of Bialystok, 15-089 Bialystok, Poland; ladnyjr@wp.pl

**Keywords:** COVID-19, body mass index, intensive care, serial biomarkers, missing data, source validation, mortality

## Abstract

**Background/Objectives:** The association of body mass index (BMI) with outcomes after intensive care unit (ICU) admission remains uncertain. We examined ICU mortality and early biomarker patterns in adults with COVID-19. **Methods:** This single-center retrospective secondary analysis included 201 patients. BMI was analyzed per 5 kg/m^2^ using logistic regression for mortality and generalized estimating equations for biomarkers, adjusted for age, sex, and calendar time. Six inflammatory and coagulation biomarkers were averaged within four consecutive 24 h intervals covering the first 96 h after ICU admission. False-discovery-rate correction and missingness sensitivity analyses were performed. **Results:** Eighty patients died (39.8%). The adjusted mortality odds ratio was 1.02 (95% CI 0.82–1.26). Joint tests of interval-specific BMI associations were significant for interleukin-6 (IL-6) and white blood cell count, but not the other four biomarkers under the primary specification. Only IL-6 at 24–48 h remained significant after correction across 24 contrasts (−35.9% per 5 kg/m^2^ higher BMI; 95% CI −49.4 to −18.8; q = 0.006), based on 40/201 patients. **Conclusions:** Within this selected ICU cohort, BMI showed no clear adjusted mortality association and biomarker findings were heterogeneous. The isolated inverse IL-6 association remains exploratory because of sparse measurements and potentially informative missingness and requires independent confirmation with standardized sampling.

## 1. Introduction

Higher body mass index (BMI) has been associated with adverse COVID-19 outcomes across population-based and clinical cohorts [1,2,3,4]. These studies address several distinct transitions, including infection, hospital admission, escalation of respiratory support, and death. An association with the combined risk of reaching critical illness does not imply that the same association will be observed among patients who have already been admitted to an intensive care unit (ICU). Defining the population and the stage of disease is therefore essential when interpreting BMI-related risk.

Several biological pathways make a relationship between adiposity and severe infection plausible. These include altered immune responses, chronic inflammation, impaired respiratory mechanics, endothelial dysfunction, and thrombosis [5,6,7]. BMI is nevertheless an indirect measure. It does not distinguish adipose tissue from muscle, identify visceral fat, or capture metabolic health. Acute changes in fluid balance can also influence admission weight. Consequently, a BMI coefficient should not be interpreted as a direct estimate of the biological effect of adipose tissue.

Studies examining inflammatory markers have reported heterogeneous findings [8,9,10,11]. Differences in the population, timing and frequency of blood sampling, treatment exposure, and statistical adjustment may contribute to this variation. A further difficulty is that clinically ordered biomarker measurements are not a scheduled experimental series. A missing IL-6 result may reflect a decision not to test, clinical improvement, transfer, or death. Comparing only observed results can therefore select different patients at successive time points.

Selection into intensive care creates another interpretative constraint. Both BMI-related disease processes and other determinants of prognosis can affect ICU admission. Conditioning on this shared consequence can induce an association between otherwise unrelated factors and distort the association between BMI and mortality [12,13,14]. A weak association within an ICU cohort can coexist with a harmful population-level association. It neither demonstrates an obesity paradox nor establishes that BMI has no clinical relevance.

Respiratory severity is relevant to this distinction. The arterial oxygen partial pressure to inspired oxygen fraction ratio (PaO_2_/FiO_2_) and alveolar–arterial oxygen gradient describe overlapping aspects of gas exchange [15]. These physiological measurements and APACHE II scores may be downstream of processes associated with BMI. Including them in a regression changes the conditional association being estimated. We therefore distinguish a parsimonious demographic and calendar-time adjustment from sensitivity models that additionally condition on acute severity or treatment.

This study revisits a previously reported single-center cohort [16] with BMI as the principal exposure. The objectives are to estimate the association between admission BMI and ICU mortality within this selected population and to describe BMI-related differences in early serial inflammatory and coagulation measurements. The biomarker analyses are exploratory secondary analyses; they were not prospectively registered, and the analysis was developed with knowledge of the earlier results.

## 2. Materials and Methods

### 2.1. Study Design and Setting

This retrospective secondary analysis concerns adults treated in the ICU of the University Clinical Hospital in Bialystok, Poland, a tertiary-care university referral hospital. The original cohort publication describes a screening period from 3 March 2020 to 1 July 2021 [16]. Included patients were admitted between 25 September 2020 and 19 June 2021; the ICU first admitted patients with critical COVID-19 in September 2020. The itemized historical screening log was unavailable. Reporting follows the STROBE framework [17].

### 2.2. Participants and Relationship to the Earlier Report

**Figure 1:**
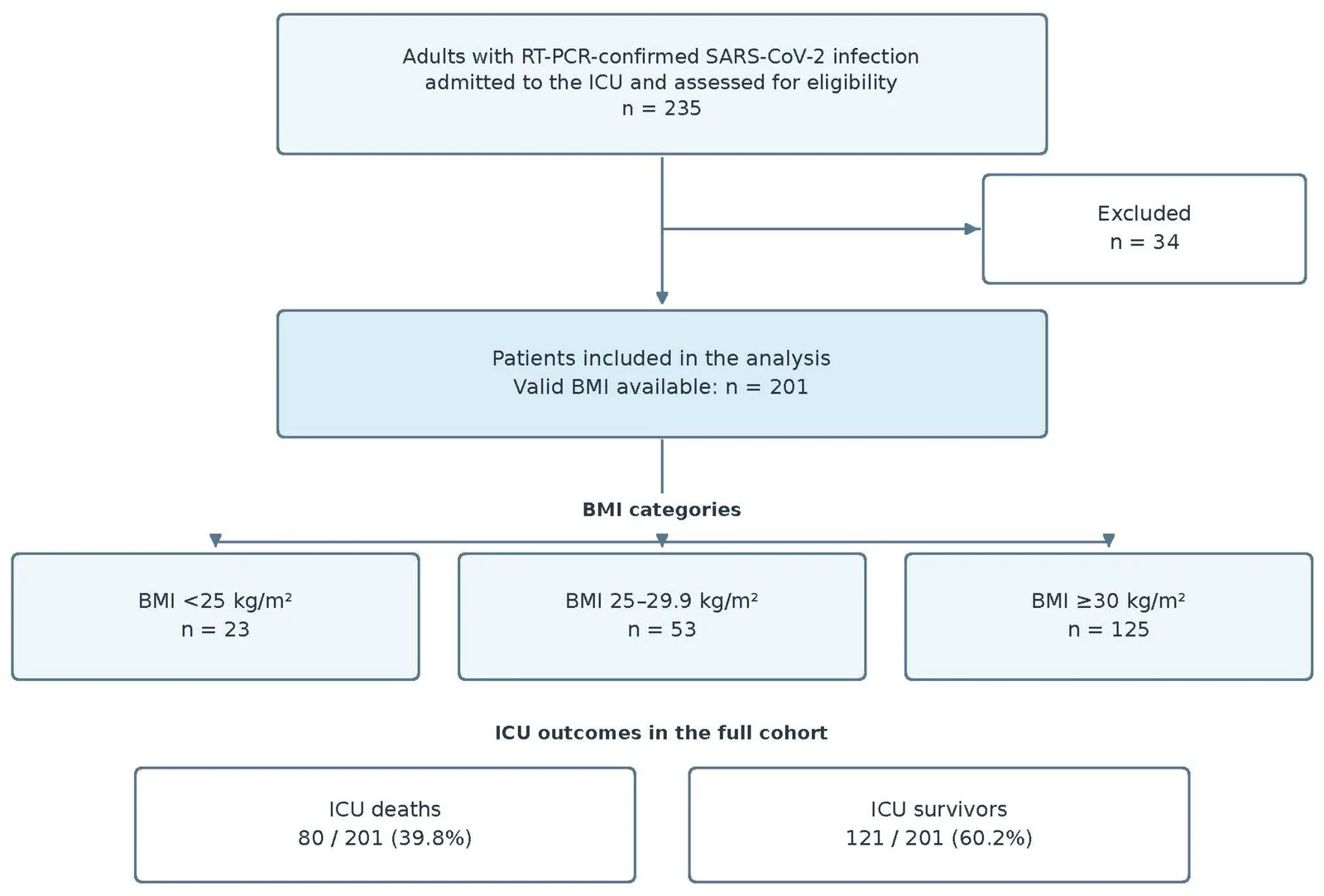
Cohort selection and BMI categories. ICU outcomes comprised 80 deaths and 121 survivors. Screening and exclusion counts derive from the earlier report [16]; BMI categories reflect the current analysis.

Eligibility in the original study required an age of at least 18 years, acute SARS-CoV-2 infection confirmed by reverse transcription polymerase chain reaction, and ICU admission primarily for COVID-19. Pregnancy and admission for another primary indication were exclusion criteria. The original study reported 235 screened patients, 34 exclusions, and 201 included patients [16]. The present analysis retains all 201 patients, irrespective of biomarker availability. Figure 1 reports the published selection counts and BMI distribution; separate exclusion counts by reason could not be reconstructed from the available records.

The original PLoS ONE report describes this same 201-patient cohort, including 80 deaths and 121 survivors, within the same published recruitment window [16]. It compared BMI by survival status and included BMI in a univariable mortality analysis. Its WBC and IL-6 comparisons were between survival groups; no BMI–WBC comparison was identified in its published text or tables. The present secondary analysis addresses BMI as a continuous exposure and models serial biomarker concentrations using patient-level clustering. It examines a distinct analytical question in the same patients and does not constitute an independent cohort.

### 2.3. Data Sources and Validation

Clinical data were obtained from the hospital information system and medical records. The main registry comprised 201 patients; laboratory records were linked to 198 patients, and clinical narrative and TISS data were available for 196 patients. The laboratory dataset contained 102,153 records. Data were linked using study identifiers, with admission and discharge dates checked for consistency. Patient-level coverage and usable observations are reported in Supplementary Table S11.

The investigators checked laboratory values against direct extracts from the hospital information system. Exact measurements were distinguished from censored and nonnumeric results, and records outside the index ICU stay were excluded. Specimen-collection times defined the laboratory intervals described in Section 2.6.

### 2.4. BMI Definition and Anthropometry

Height and weight were measured at ICU admission, and BMI was calculated as weight in kilograms divided by squared height in meters. The values were not self-reported. Details of the weighing equipment and the timing relative to fluid administration were unavailable in the records.

BMI was analyzed continuously per 5 kg/m^2^. Categories of <25, 25 to <30, and ≥30 kg/m^2^ were used for description and sensitivity analysis, based on conventional thresholds [18]. Underweight and higher obesity classes were not used as separate inferential groups because the available cohort would produce small, imprecise comparisons. The three groups contain 23, 53, and 125 patients, respectively. Recorded admission BMI values were checked against the available source anthropometric measurements.

### 2.5. ICU Mortality Outcome

The outcome was death during the index ICU admission. Outcomes for all 201 patients were verified against the hospital’s internal outcome report, discharge summaries, and medical records, confirming 80 ICU deaths and 121 ICU survivors.

### 2.6. Laboratory Measures and Time Windows

The biomarkers were CRP (mg/L), D-dimer (µg/mL as recorded), INR (dimensionless), IL-6 (pg/mL), PCT (ng/mL), and WBC (10^9^/L). The D-dimer FEU/DDU convention, reagent-specific detection limits, and complete assay metadata were unavailable; no unit conversion was performed. The original publication reports single-laboratory testing, immunoassay for PCT, ELISA for IL-6, turbidimetry for CRP, and automated hematology analysis [16]. These assay descriptions are attributed to that report.

Laboratory results were assigned to intervals according to specimen-collection date and time. Exact positive results were averaged within consecutive intervals of [0,24), [24,48), [48,72), and [72,96) hours after ICU admission, restricted to the recorded ICU stay. These were labeled day 0, day 1, day 2, and day 3. They summarize samples collected within each interval, rather than scheduled blood draws at admission, 24, 48, or 72 h. The fourth interval extends to 96 h.

Laboratory testing was clinically ordered. Samples collected after ICU discharge were excluded. In the censoring sensitivity analysis, <L or ≤L values were replaced by L and >U or ≥U values by U before arithmetic interval averaging and log_2_ transformation; censored values were excluded from the primary means.

### 2.7. Clinical Covariates, Treatments, and TISS

Core covariates were age, sex, admission calendar time, and APACHE II. Additional sensitivity covariates included documented diabetes, hypertension, heart failure, chronic organ insufficiency or immune compromise, one respiratory severity measure at a time, and source indicators for corticosteroid treatment and mechanical ventilation. Whole-stay treatment indicators were not assumed to represent exposure before a particular laboratory measurement. They were omitted from the primary model because they may occur after the exposure, evolve with disease severity, or lie on pathways associated with BMI.

Clinical narratives, recorded comorbidities, medication records, and diagnostic codes were reviewed for additional diagnoses, immunomodulatory and antiviral treatment, anticoagulation, and vaccination. Positive documentation was recorded separately from unknown status; absence of documentation was not classified as absence of disease or treatment. Variables without reliable treatment documentation were not used as additional adjustment covariates. TISS provided whole-stay intervention counts for 196 patients, including vasoactive therapy and renal replacement therapy. These counts support descriptive reporting but do not establish treatment timing before each biomarker measurement.

SARS-CoV-2 variants were not routinely determined and were not inferred from admission dates. Individual vaccination information was incomplete. The study period overlapped the introduction of COVID-19 vaccination in Poland on 27 December 2020 [19]. Documented vaccination was recorded, whereas status remained unknown in patients without such documentation.

### 2.8. Target Associations and Statistical Models

The primary estimand is an ICU-conditional association: the odds ratio for ICU death per 5 kg/m^2^ higher BMI after adjustment for age, sex, and calendar time among the included patients. It is not a population total effect, a controlled direct causal effect, or a validated prediction model. A conceptual diagram in Figure S1 illustrates the selection mechanism and why adjustment for admission severity or treatment does not necessarily remove bias. Residual confounding and conditioning on ICU admission remain even in the parsimonious model.

Continuous variables are summarized as median [interquartile range], and categorical variables as *n*/*N* (%), with denominators reflecting observed source data. Baseline differences were summarized using standardized differences. For three BMI groups, the maximum absolute pairwise standardized difference is reported; this is descriptive and was not used to select regression variables.

Logistic regression models compare the unadjusted estimate, the primary age/sex/calendar estimate, addition of APACHE II, and further severity or treatment adjustment. PaO_2_/FiO_2_ and the alveolar–arterial gradient are evaluated in separate sensitivity models because they contain overlapping respiratory information, and APACHE II already incorporates acute physiology. Each model reports its effective N, events, number of slopes and total coefficients including the intercept. Expanded models are sensitivity analyses, not evidence that increasingly extensive adjustment recovers a causal effect. Calendar time is represented as elapsed time per approximately three months, with categorical-period sensitivity analyses. A three-knot restricted cubic spline evaluates possible BMI nonlinearity; its curve, confidence bands, and exposure distribution are shown in the supplement. Interaction analyses, where reported, are explicitly exploratory.

Mortality model stability was assessed with 1000 patient-level bootstrap resamples using a fixed random seed and unchanged predictor sets. Empirical BMI coefficient intervals, apparent and optimism-corrected AUC, and calibration slopes were calculated. These diagnostics assess statistical stability and prediction performance within the available cohort; they do not establish external predictive validity. Convergence and collinearity diagnostics were also recorded.

### 2.9. Serial Biomarker Models and Missing Data

Each patient’s arithmetic within-interval biomarker mean was log_2_-transformed. Gaussian generalized estimating equation (GEE) models used exchangeable within-patient correlation and robust sandwich standard errors [20]. The parsimonious model includes BMI, categorical interval, their interaction, age, sex, and calendar time. Back-transformed contrasts are percentage differences per 5 kg/m^2^ higher BMI, calculated as 100 × (2^β^ − 1), where β is the relevant interval-specific coefficient or linear contrast. These are between-patient differences in modeled interval summaries, not within-patient changes over time. A global BMI test is the joint four-degree-of-freedom test of the BMI main coefficient and three interaction coefficients; it is not a single effect averaged over time. The three-degree-of-freedom interaction test evaluates variation between intervals. Estimated correlations, unique patients, total observations, and the number of measurements per patient are reported.

Benjamini–Hochberg false-discovery-rate adjustment is applied separately to six global BMI tests, six interaction tests, and 24 interval-specific contrasts [21]. The interval-specific family includes every marker and interval, regardless of nominal significance. Displayed 95% confidence intervals are pointwise and are not multiplicity-adjusted. Multiplicity correction does not correct observation bias, confounding, endpoint errors, or incorrect sampling-time definitions.

No unmeasured biomarker was automatically assigned a normal value, and no undocumented comorbidity or treatment was assigned “No”. The primary models use available exact positive results and complete adjustment covariates. Ordinary unweighted GEE is not generally valid under arbitrary missing-at-random mechanisms. We therefore describe observation counts, patients with complete profiles, characteristics of measured and unmeasured subsets, and associations with available outcome and severity indicators. Missing measurements after discharge are distinguished from the absence of a test while still in the ICU wherever the recorded dates permit.

Sensitivity analyses evaluate an independence working correlation, restriction to patients with repeated IL-6 measurements, and inverse-probability-of-observation weighting [22]. Observation weighting addresses only measured determinants under a correctly specified observation model and adequate positivity. It cannot establish that testing was non-informative, correct missingness depending on an unobserved biomarker, or recover measurements after death. Separate logistic observation models for each marker and interval used BMI, age, sex, APACHE II, and calendar time; follow-up intervals additionally used the baseline log_2_ marker and a baseline-missing indicator. Missing baseline marker predictors were replaced by the marker median solely in the observation model, not as outcome measurements. Stabilized weights were the observed interval fraction divided by the predicted observation probability and were capped at the 1st and 99th percentiles. The weighted GEE used independence working correlation and a robust variance conditional on the estimated weights; it did not propagate all weight-estimation uncertainty. Weight diagnostics are reported in Supplementary Table S7. Informative testing and residual confounding remain limitations; sensitivity analyses do not establish complete bias correction.

### 2.10. Reproducibility

Analysis code, a statistical workbook, and nonidentifying aggregate outputs accompany the Supplementary Materials. Re-execution requires the restricted patient-level source data specified in the accompanying documentation. Direct identifiers and clinical narratives are not publicly shared.

## 3. Results

### 3.1. Cohort and BMI

Admission BMI was available for all 201 patients. Median BMI was 31.0 [27.3–35.0] kg/m^2^ (range, 17.0–56.0). There was one underweight patient, 22 with BMI 18.5 to <25, 53 with BMI 25 to <30, 74 with BMI 30 to <35, 31 with BMI 35 to <40, and 20 with BMI ≥ 40 kg/m^2^. The three analytical categories were therefore 23, 53, and 125 patients. The ICU mortality was 80/201 (39.8%). Table 1 separates observed denominators from missing values. Patients with BMI ≥ 30 had a lower median PaO_2_/FiO_2_ ratio than the two lower categories (96.0 versus 120.0 and 124.7 mmHg); the maximum absolute pairwise standardized difference was 0.450. These comparisons are descriptive, without baseline significance testing.

### 3.2. BMI and ICU Mortality

**Figure 2:**
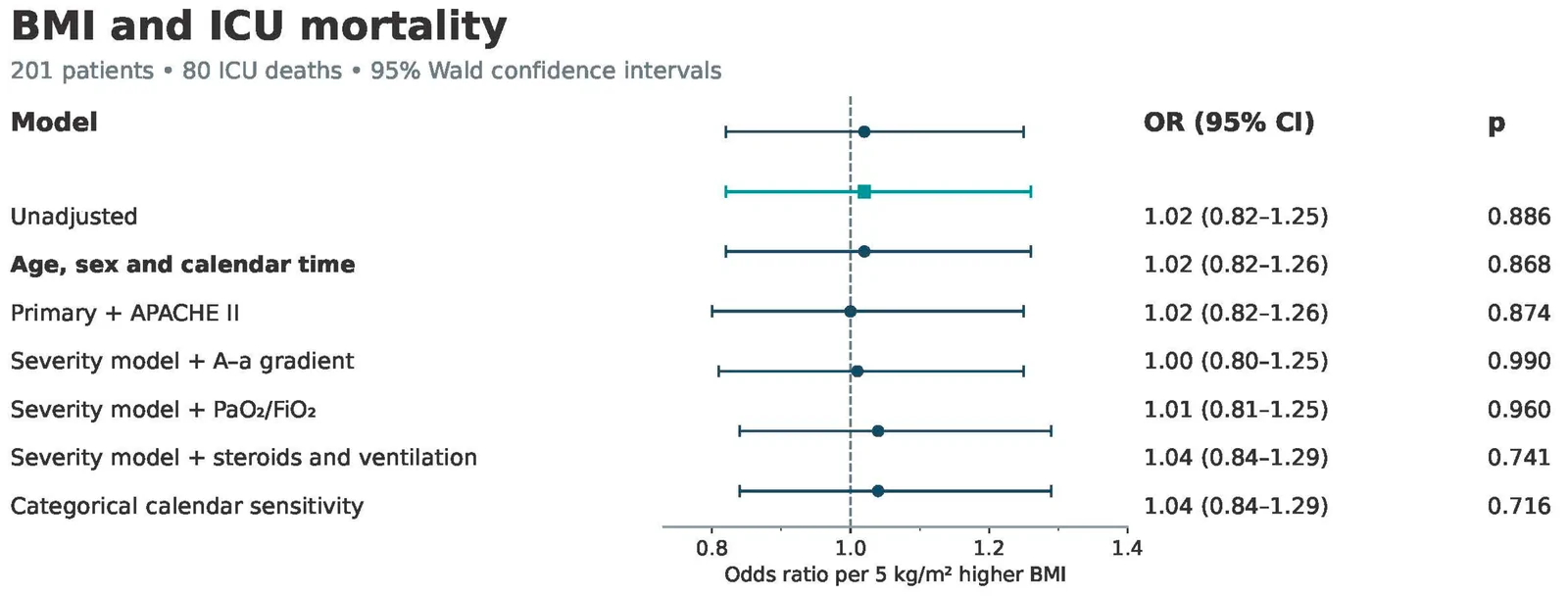
Associations between BMI and ICU mortality. Points and bars indicate odds ratios per 5 kg/m^2^ higher BMI and 95% confidence intervals. The vertical dashed line indicates the null value (OR = 1). The teal marker and confidence interval identify the primary age-, sex-, and calendar-time adjusted model; the remaining estimates represent sensitivity analyses. All seven displayed models include 201 patients and 80 deaths.

The unadjusted OR per 5 kg/m^2^ higher BMI was 1.02 (95% CI 0.82–1.25; *p* = 0.886). The age-, sex-, and calendar-adjusted OR was 1.02 (95% CI 0.82–1.26; *p* = 0.868), using all 201 patients and 80 deaths. The primary model estimated four slopes plus an intercept, corresponding to 20 events per slope. Addition of APACHE II gave an OR of 1.02 (95% CI 0.82–1.26; *p* = 0.874). Table 2 and Figure 2 provide sequential model estimates. The extended sensitivity model used 199 patients and 79 deaths, with ten slopes; its BMI OR was 0.99 (95% CI 0.78–1.24; *p* = 0.902). Category estimates were imprecise: 1.00 (95% CI 0.36–2.78) for BMI 25 to <30 versus <25, and 0.97 (95% CI 0.39–2.43) for BMI ≥ 30 versus <25.

All 1000 bootstrap fits of the primary model converged. The empirical BMI OR interval was 0.81–1.26. Predictive-performance diagnostics do not support clinical prediction use (Supplementary Table S10). The restricted cubic spline nonlinearity likelihood-ratio *p* value was 0.312; wide confidence bands at sparsely populated BMI values remain compatible with alternative shapes (Figure S2). Exploratory interaction estimates and full model coefficients are reported in Supplementary Table S3 and the statistics workbook.

### 3.3. Serial Biomarker Analyses

The primary biomarker analysis included 2563 patient–marker–interval observations. CRP contributed 580 observations from 196 patients, D-dimer 239 from 127, INR 392 from 194, IL-6 245 from 149, PCT 552 from 195, and WBC 555 from 196. In the six-marker global-test family, IL-6 (q < 0.001) and WBC (q = 0.002) showed model-based BMI-related variation; CRP (q = 0.651), D-dimer (q = 0.384), INR (q = 0.384), and PCT (q = 0.651) did not. BMI-by-interval interaction q values were 0.002 for IL-6 and 0.001 for WBC (Table 3).

**Figure 3:**
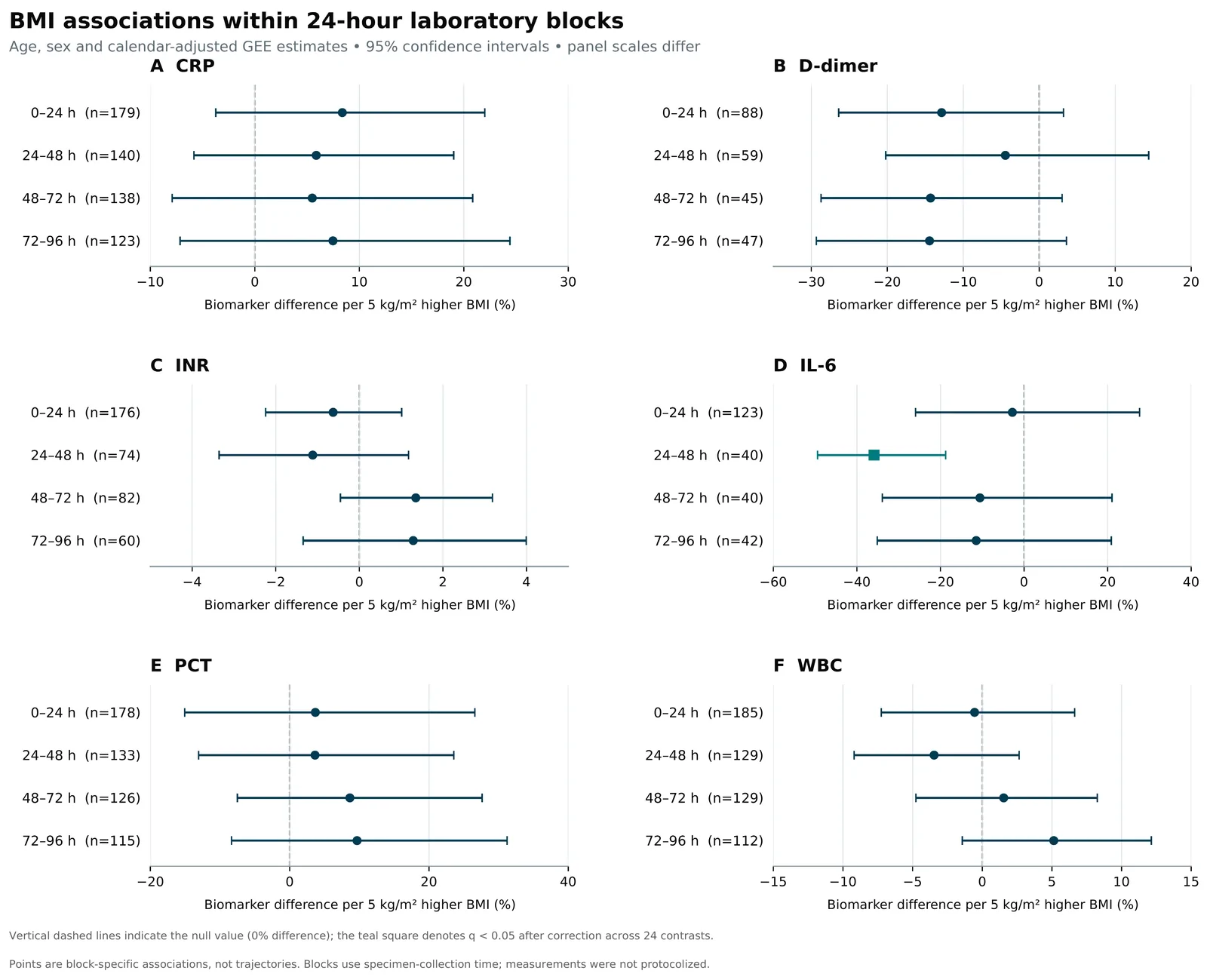
Interval-specific biomarker associations per 5 kg/m^2^ higher BMI: (**A**) C-reactive protein (CRP); (**B**) D-dimer; (**C**) international normalized ratio (INR); (**D**) interleukin-6 (IL-6); (**E**) procalcitonin (PCT); and (**F**) white blood cell count (WBC). Points and bars indicate percentage differences and pointwise 95% confidence intervals, which are not multiplicity-adjusted. The teal square identifies the IL-6 24–48 h contrast that remained significant after false-discovery-rate correction across all 24 interval-specific contrasts. The vertical dashed line indicates the null value (0% difference). Results are interval means based on specimen-collection time and do not represent measurements from a fixed-time blood-draw schedule.

Among all 24 interval-specific contrasts, only IL-6 in the 24–48 h block met the FDR threshold: −35.9% (95% CI −49.4 to −18.8) per 5 kg/m^2^ higher BMI; *p* < 0.001; q = 0.006. This was based on 40/201 patients (19.9%). It is an association with a within-interval summary among available measurements, not a verified IL-6 effect at exactly 24 h. No WBC interval-specific confidence interval excluded zero, despite the significant joint tests. These tests address different hypotheses, and the joint statistic incorporates covariance among the interval-specific estimates. Complete effect estimates, *p* values and q values are shown in Figure 3 and Supplementary Table S2.

### 3.4. Observation Patterns and Sensitivity Analyses

Only seven patients had all four IL-6 summaries and nine had all four D-dimer summaries. For IL-6, 52 patients had no exact positive value, 85 had one interval, 39 had two, and 18 had three. The 40 patients with IL-6 in the 24–48 h block had median BMI 31.4 [28.9–34.1] kg/m^2^ (range, 21.0–55.0); the BMI-category counts were 4, 9, and 27. APACHE II was 29.5 [20.8–32.5] in the measured subset versus 31.0 [22.0–35.0] in those without that interval measurement (absolute standardized difference, 0.257). Whole-stay corticosteroid documentation was 95.0% versus 86.3% (standardized difference, 0.301). These comparisons show why similar measurement fractions by BMI would not establish non-informative observation. Table 4 and Supplementary Tables S4 and S5 report availability and measured-subset characteristics.

For the IL-6 24–48 h contrast, observation weighting gave −48.4% (95% CI −65.2 to −23.7), *p* < 0.001, with an effective weighted sample size of 31.0 among 40 measured patients. The corresponding unweighted independence-correlation estimate was −49.9% (95% CI −60.9 to −35.7), indicating that the difference from the primary exchangeable-correlation estimate should not be attributed to weighting alone. Restriction to patients with at least two IL-6 measurements gave −43.1% (95% CI −55.6 to −27.0) (34 measurements in the interval, 64 patients overall). Excluding 18 patients with documented tocilizumab produced −38.1% (95% CI −51.0 to −21.9) (37 interval measurements); the remaining patients were not assumed to be untreated. Bias-reduced sandwich and independence-correlation results are in Supplementary Table S6; an actual-time-gap autoregressive fit failed for five of six markers and was not interpreted as a completed family of analyses. A model restricted to seven complete IL-6 profiles was not fitted because it would estimate eleven coefficients from seven clusters.

### 3.5. Additional Clinical Characteristics

Clinical records documented COVID-19 vaccination in 5 patients, tocilizumab in 18, remdesivir in 17, and chronic kidney disease in 22; 20 patients had an N18 diagnostic code. These are documentation counts, with unknown status for patients without documentation, rather than complete exposure-prevalence estimates. TISS comprised 2764 entries across 196 patients. Positive whole-stay counts indicated mechanical ventilation in 180/196, a single vasoactive drug in 167/196, multiple vasoactive drugs in 128/196, either vasoactive category in 183/196, and renal replacement therapy in 36/196. The timing needed for baseline or pre-biomarker adjustment could not be established (Supplementary Table S9).

Fifty-eight censored laboratory results were recorded within the four-interval observation window and were evaluated separately in sensitivity analyses.

## 4. Discussion

### 4.1. Principal Findings and Their Scope

In this cohort of 201 critically ill adults with COVID-19, admission BMI was not clearly associated with ICU mortality after adjustment for age, sex, and calendar time. The estimate was close to one, but its confidence interval remained compatible with clinically relevant differences. Biomarker analyses did not identify a uniform inflammatory or coagulation pattern across BMI. IL-6 and WBC had significant global tests, whereas only the IL-6 24–48 h contrast survived correction across individual intervals. That finding was based on 40 patients; sparse, clinically determined observation and incomplete treatment chronology limit its biological interpretation.

The analyses estimate associations within a selected ICU population. A confidence interval spanning the null does not establish equivalence or exclude effects that may matter clinically. It is also compatible with BMI influencing the probability of hospitalization or ICU admission before the stage of illness observed here. The results cannot justify describing elevated BMI as protective or asserting an obesity paradox [12,13,14].

### 4.2. Comparison with Previous Research

Large population and healthcare-based studies have linked higher BMI with severe COVID-19 outcomes [1,2,3,4]. Their findings and an imprecise within-ICU association address different populations and clinical transitions. Similarly, hospitalized-patient studies of inflammation should not be expected to reproduce the same biomarker pattern in a cohort restricted to critical illness. McNeill and colleagues reported differences in CRP, ESR, and D-dimer but not a uniform elevation of every inflammatory marker [8]. Other reports describe heterogeneous biomarker relationships and analyses of systemic inflammation in the association between obesity and adverse outcomes [9,10,11].

Such literature provides context rather than external validation of the current coefficient estimates. The sampling strategy, assay handling, disease stage, and treatment all affect measured concentrations. In particular, IL-6 values after immunomodulation cannot be interpreted as an untreated inflammatory phenotype. An association in a small clinically selected, measured subset does not demonstrate that BMI directly modifies cytokine production. FDR adjustment limits one form of multiplicity-related error but does not make a selected or incorrectly timed measurement representative.

### 4.3. Treatment Timing and Analytical Sensitivity

Whole-stay TISS counts cannot establish whether treatment preceded an IL-6 assay. A diagnosis recorded later during admission may not represent a baseline comorbidity. Incorporating such variables without accounting for their timing could increase bias despite apparently more extensive adjustment.

The D-dimer joint test was sensitive to working-correlation specification (*p* = 0.227 under exchangeability versus *p* = 0.0017 under independence; Supplementary Table S6B).

### 4.4. Strengths and Limitations

The study retains the full 201-patient cohort, treats BMI continuously, uses parsimonious primary adjustment, accounts for within-patient correlation, and distinguishes multiple-testing families. Source verification and detailed reporting of biomarker availability support interpretation of the findings.

The study remains retrospective, single-center, and observational. The BMI distribution is unbalanced, with only 23 patients below 25 kg/m^2^, and estimates for BMI categories or extreme values are imprecise. Admission BMI does not capture body composition or chronic weight history. Despite source verification, residual BMI measurement error and category misclassification may affect the estimated associations. Acute severity and treatments may be intermediates, and conditioning on ICU admission can create selection bias that regression within the ICU cohort cannot remove.

Biomarker observation is incomplete and clinically determined. Patients contributing repeated results may differ from patients with one or no result, and ordinary GEE robustness to correlation misspecification does not imply robustness to informative missingness. Observation weighting is dependent on measured predictors and model specification. Sparse IL-6 subsets weaken the practical support for asymptotic inference, and neither weighting nor correlation sensitivity establishes a missing-not-at-random mechanism. Censored laboratory results and incomplete assay metadata impose additional limits. Variant data are unavailable, vaccination and several treatments are incompletely documented, and linear or categorical calendar adjustment cannot fully represent all changes in pandemic care [23]. Collection timestamps define interval membership, but clinically ordered tests and interval averages do not constitute a fixed sampling schedule. Inference about biological changes at exact post-admission times therefore remains limited, particularly for IL-6.

### 4.5. Implications and Future Research

Interpretation of secondary ICU analyses requires a clearly defined population and a distinction between baseline covariates and measurements or treatments arising during critical illness. The present findings do not support BMI-specific thresholds for IL-6 testing, a change in immunomodulatory treatment, or a clinical prediction score. The mortality models were designed to examine an association and have not been externally validated for individual prognosis.

A stronger future study would prospectively standardize sampling schedules, retain assay units and censoring limits, document treatments before each blood draw, and link verified ICU outcomes to stable patient and admission identifiers. Repeated anthropometry or body-composition measures would help distinguish BMI from adiposity. Multicenter data with broader BMI representation and structured follow-up of testing decisions would improve precision and permit sensitivity analyses for informative observation. External validation should use an independent cohort rather than another analysis of these same patients.

## 5. Conclusions

No clear adjusted association between admission BMI and ICU mortality was observed in this selected cohort. The confidence interval does not establish equivalence across BMI values. Serial biomarker associations were heterogeneous, and the exploratory IL-6 finding was based on a small, incompletely observed subset. These results do not establish an obesity paradox, a causal protective effect of higher BMI, or a basis for BMI-directed treatment.

## Figures and Tables

**Table 1:** Characteristics by BMI category.

Characteristic	Overall *N* = 201	<25 *N* = 23	25 to <30 *N* = 53	≥30 *N* = 125	Max. |SMD|
Age, years	67.0 [60.0–73.0]	62.0 [51.0–70.0]	69.0 [64.0–76.0]	66.0 [59.0–72.0]	0.601
BMI, kg/m^2^	31.0 [27.3–35.0]	23.0 [21.9–24.0]	27.2 [26.0–28.7]	33.7 [31.0–37.8]	2.902
APACHE II score	30.0 [22.0–35.0]	34.0 [24.0–37.0]	29.0 [22.0–35.0]	30.0 [22.0–34.0]	0.263
Glasgow Coma Scale	3.0 [3.0–15.0]	3.0 [3.0–13.0]	3.0 [3.0–13.0]	3.0 [3.0–15.0]	0.103
PaO_2_/FiO_2_, mmHg	101.3 [72.4–157.8]	120.0 [94.4–174.3]	124.7 [82.5–175.7]	96.0 [70.4–129.0]	0.450
Alveolar–arterial gradient, mmHg	501.8 [319.1–609.2]	352.2 [260.3–565.9]	410.2 [245.6–604.9]	531.8 [384.6–619.4]	0.496
Female sex	87/201 (43.3%)	10/23 (43.5%)	21/53 (39.6%)	56/125 (44.8%)	0.105
Diabetes mellitus	60/201 (29.9%)	5/23 (21.7%)	11/53 (20.8%)	44/125 (35.2%)	0.326
Atrial fibrillation	28/201 (13.9%)	7/23 (30.4%)	2/53 (3.8%)	19/125 (15.2%)	0.757
Hypertension	121/200 (60.5%)	7/22 (31.8%)	32/53 (60.4%)	82/125 (65.6%)	0.718
Congestive heart failure	46/200 (23.0%)	5/23 (21.7%)	13/53 (24.5%)	28/124 (22.6%)	0.066
Chronic organ insufficiency/immune compromise	128/201 (63.7%)	16/23 (69.6%)	25/53 (47.2%)	87/125 (69.6%)	0.467
Corticosteroids, any time	177/201 (88.1%)	19/23 (82.6%)	46/53 (86.8%)	112/125 (89.6%)	0.203
Mechanical ventilation, any time	183/201 (91.0%)	20/23 (87.0%)	47/53 (88.7%)	116/125 (92.8%)	0.195
Neuromuscular blocking agents	135/201 (67.2%)	11/23 (47.8%)	30/53 (56.6%)	94/125 (75.2%)	0.586
Prone positioning	79/201 (39.3%)	7/23 (30.4%)	15/53 (28.3%)	57/125 (45.6%)	0.364
Antibiotics	182/201 (90.5%)	22/23 (95.7%)	49/53 (92.5%)	111/125 (88.8%)	0.258
ICU death	80/201 (39.8%)	9/23 (39.1%)	22/53 (41.5%)	49/125 (39.2%)	0.049

BMI units are kg/m^2^. Continuous variables are median [IQR]; binary variables are n/N (%). All continuous entries have the group N stated in the header. Binary denominators reflect missing source values. SMD is the largest absolute pairwise standardized difference. Treatment indicators describe any recorded exposure during the stay, not necessarily admission treatment. APACHE II, Acute Physiology and Chronic Health Evaluation II; ICU, intensive care unit; PaO_2_/FiO_2_, arterial oxygen pressure/inspired oxygen fraction.

**Table 2:** Associations between BMI and ICU mortality.

Model/Contrast	N/Events	Total Coefficients	OR (95% CI)	*p*
Unadjusted	201/80	2	1.02 (0.82–1.25)	0.886
Primary age/sex/calendar	201/80	5	1.02 (0.82–1.26)	0.868
Severity conditional: primary + APACHE II	201/80	6	1.02 (0.82–1.26)	0.874
Respiratory sensitivity: APACHE + A–a gradient	201/80	7	1.00 (0.80–1.25)	0.990
Respiratory sensitivity: APACHE + P/F ratio	201/80	7	1.01 (0.81–1.25)	0.960
Treatment conditional: severity + steroids/ventilation	201/80	8	1.04 (0.84–1.29)	0.741
Calendar categorical sensitivity	201/80	6	1.04 (0.84–1.29)	0.716
Extended adjustment	199/79	11	0.99 (0.78–1.24)	0.902
BMI category 25 to <30 vs. <25	201/80	6	1.00 (0.36–2.78)	0.995
BMI category ≥30 vs. <25	201/80	6	0.97 (0.39–2.43)	0.944

Continuous effects are per 5 kg/m^2^ higher BMI. Total coefficients include the intercept. The primary model has four slopes; APACHE II is added only in sensitivity models. Expanded adjustment includes diabetes, hypertension, heart failure, chronic organ insufficiency/immune compromise and the A–a gradient. Category models use BMI < 25 as reference. Model-specific event counts reflect complete-case denominators; the whole cohort contains 80 deaths.

**Table 3:** Joint BMI and BMI-by-interval tests for biomarkers.

Marker	Observations/Patients	ρ	Global *p*	Global q	Interaction *p*	Interaction q
CRP	580/196	0.760	0.641	0.651	0.903	0.903
D-dimer	239/127	0.708	0.227	0.384	0.508	0.762
INR	392/194	0.672	0.256	0.384	0.156	0.313
IL-6	245/149	0.714	<0.001	<0.001	<0.001	0.002
PCT	552/195	0.850	0.651	0.651	0.721	0.865
WBC	555/196	0.757	<0.001	0.002	<0.001	0.001

Gaussian GEE models use log_2_ interval means, exchangeable correlation and robust sandwich variance, adjusted for age, sex and calendar time. Each estimates 11 coefficients including the intercept. The global test jointly tests four BMI-related coefficients; the interaction test tests three coefficients. Separate Benjamini–Hochberg families each contain six tests. ρ is estimated within-patient correlation. Intervals are defined by specimen-collection time.

**Table 4:** Available exact positive biomarker summaries by interval.

Marker	0–24 h	24–48 h	48–72 h	72–96 h	Four Intervals
CRP	179	140	138	123	61
D-dimer	88	59	45	47	9
INR	176	74	82	60	11
IL-6	123	40	40	42	7
PCT	178	133	126	115	49
WBC	185	129	129	112	56

Counts refer to unique patients, out of 201 included patients. Intervals are left-inclusive and right-exclusive. Interval assignment uses specimen-collection time and excludes results obtained after ICU discharge. Nine, 13 and 14 patients had exited before the start of the last three intervals, respectively. Missingness includes censored-only and unmeasured results; no missing result is replaced by normality.

## Data Availability

The earlier cohort publication provides supporting data [16]. Analysis code, the statistics workbook, and nonidentifying aggregate outputs are supplied in JCM_4529624_Reproducibility.zip. Patient-level source data and clinical narratives require institutional authorization and appropriate protection of patient confidentiality.

## Supplementary Materials

The following supporting information can be downloaded at: https://www.mdpi.com/article/10.3390/jcm15207799/s1, Table S1. Early biomarker distributions by admission BMI category; Table S2. Complete primary interval-specific GEE estimates; Table S3. Mortality sensitivity and interaction estimates; Table S4. Observation availability and longitudinal profiles; Table S5. Characteristics of patients with and without an observed IL-6 value; Table S6A. IL-6 24–48-hour sensitivity estimates; Table S6B. Joint BMI tests under alternative working correlations; Table S7. Inverse-probability-of-observation diagnostics; Table S8. Calendar-period description; Table S9. Additional clinical documentation and TISS; Table S10. Bootstrap and spline diagnostics; Table S11. Clinical cohort, source linkage, and analytical coverage; Figure S1. Conceptual relationships and ICU selection; Figure S2. Exploratory BMI spline with pointwise 95% confidence band and the observed exposure distribution; Figure S3. Sensitivity estimates for the IL-6 24–48-hour interval; analysis code and aggregate statistical outputs.
