# Supplementary material for: Association of Body Mass Index with Early Inflammatory and Coagulation Biomarker Patterns and Intensive Care Unit Mortality in Critically Ill Patients with COVID-19: A Retrospective Cohort Study
Source: J Clin Med. 2026 Oct 9;15(20):7799. doi: 10.3390/jcm15207799 (PMC13705132; doi:10.3390/jcm15207799)
Supplement: Supplementary file 1 [file jcm-15-07799-s001.zip › jcm-4529624-supplementary.pdf]

Supplementary Materials

Table S1. Early biomarker distributions by admission BMI category

Biomarker and unit	Interval	Overall	BMI <25	BMI 25 to <30	BMI ≥30
CRP (mg/L)	0–24 h	71.0 [24.8–149.9] (n=179)	68.5 [27.1–152.5] (n=20)	55.3 [18.8–129.5] (n=47)	90.8 [31.6–157.2] (n=112)
CRP (mg/L)	24–48 h	58.4 [22.0–122.7] (n=140)	50.6 [25.9–129.7] (n=16)	50.2 [10.2–131.2] (n=32)	62.6 [22.6–111.5] (n=92)
CRP (mg/L)	48–72 h	43.2 [12.5–84.8] (n=138)	41.8 [16.5–122.5] (n=12)	46.6 [9.9–83.2] (n=35)	43.7 [13.6–81.8] (n=91)
CRP (mg/L)	72–96 h	36.5 [16.2–88.8] (n=123)	52.4 [16.8–67.2] (n=14)	47.2 [18.0–138.8] (n=35)	34.2 [14.9–85.5] (n=74)
D-dimer (μg/mL)	0–24 h	1.8 [1.2–4.5] (n=88)	2.6 [1.6–12.7] (n=11)	1.9 [1.0–6.9] (n=20)	1.7 [1.1–3.5] (n=57)
D-dimer (μg/mL)	24–48 h	2.2 [1.2–8.4] (n=59)	10.4 [3.6–13.2] (n=6)	1.6 [1.0–5.8] (n=15)	2.3 [1.3–7.2] (n=38)
D-dimer (μg/mL)	48–72 h	2.0 [1.2–6.9] (n=45)	7.4 [2.0–11.0] (n=7)	1.7 [0.8–5.7] (n=13)	2.6 [1.2–5.1] (n=25)
D-dimer (μg/mL)	72–96 h	2.4 [1.4–6.3] (n=47)	7.3 [6.4–8.7] (n=4)	2.5 [2.1–8.9] (n=13)	2.2 [1.0–3.6] (n=30)
INR (ratio)	0–24 h	1.2 [1.1–1.3] (n=176)	1.2 [1.1–1.4] (n=19)	1.2 [1.1–1.3] (n=46)	1.2 [1.1–1.3] (n=111)
INR (ratio)	24–48 h	1.2 [1.1–1.3] (n=74)	1.2 [1.2–1.3] (n=11)	1.2 [1.1–1.4] (n=19)	1.2 [1.1–1.3] (n=44)
INR (ratio)	48–72 h	1.2 [1.1–1.3] (n=82)	1.2 [1.1–1.2] (n=7)	1.3 [1.1–1.4] (n=16)	1.1 [1.1–1.2] (n=59)
INR (ratio)	72–96 h	1.2 [1.1–1.2] (n=60)	1.2 [1.1–1.3] (n=9)	1.1 [1.0–1.2] (n=13)	1.1 [1.1–1.2] (n=38)
IL-6 (pg/mL)	0–24 h	114.8 [20.7–616.2] (n=123)	112.7 [25.6–1362.7] (n=15)	131.8 [26.5–546.3] (n=28)	106.3 [18.9–587.9] (n=80)
IL-6 (pg/mL)	24–48 h	257.3 [17.9–862.6] (n=40)	855.0 [512.1–1118.0] (n=4)	241.6 [107.2–330.7] (n=9)	261.1 [14.8–868.9] (n=27)
IL-6 (pg/mL)	48–72 h	212.6 [30.9–496.4] (n=40)	746.7 [470.7–1033.7] (n=4)	226.3 [80.3–391.1] (n=13)	64.1 [13.2–376.2] (n=23)
IL-6 (pg/mL)	72–96 h	124.5 [38.1–991.4] (n=42)	987.6 [484.3–1416.5] (n=4)	129.5 [60.3–745.3] (n=13)	75.7 [24.2–1053.0] (n=25)
PCT (ng/mL)	0–24 h	0.3 [0.1–0.7] (n=178)	0.6 [0.3–1.8] (n=19)	0.2 [0.1–0.6] (n=46)	0.2 [0.1–0.6] (n=113)
PCT (ng/mL)	24–48 h	0.2 [0.1–0.8] (n=133)	0.5 [0.3–1.5] (n=14)	0.2 [0.1–0.7] (n=30)	0.2 [0.1–0.6] (n=89)
PCT (ng/mL)	48–72 h	0.2 [0.1–0.6] (n=126)	0.3 [0.2–1.3] (n=12)	0.2 [0.1–0.4] (n=33)	0.2 [0.1–0.6] (n=81)
PCT (ng/mL)	72–96 h	0.2 [0.1–0.5] (n=115)	0.3 [0.2–0.8] (n=14)	0.2 [0.1–0.5] (n=33)	0.2 [0.1–0.5] (n=68)
WBC (10^9/L)	0–24 h	11.4 [7.5–17.0] (n=185)	11.2 [7.1–18.8] (n=20)	11.2 [8.7–16.5] (n=48)	11.4 [7.4–17.0] (n=117)
WBC (10^9/L)	24–48 h	12.4 [8.5–16.7] (n=129)	15.8 [9.4–19.3] (n=16)	11.8 [9.5–14.7] (n=30)	12.2 [8.3–15.8] (n=83)
WBC (10^9/L)	48–72 h	13.3 [8.5–17.2] (n=129)	14.4 [5.9–17.8] (n=13)	13.0 [9.8–15.0] (n=30)	13.3 [8.0–17.1] (n=86)
WBC (10^9/L)	72–96 h	13.4 [9.9–18.2] (n=112)	14.3 [8.6–16.5] (n=14)	12.8 [11.2–14.2] (n=32)	14.9 [9.7–20.4] (n=66)

Values are median [IQR] (available n) of patient-level arithmetic interval means. BMI categories contain 23, 53, and 125 patients. Intervals are [0,24), [24,48), [48,72), and [72,96) hours after ICU admission, defined by specimen-collection date and time. These summaries do not represent scheduled point-time sampling. D-dimer is reported in μg/mL as recorded; the FEU/DDU convention could not be verified, and external threshold comparisons are not made. WBC is expressed consistently as 10^9/L. No missing or censored result is classified as normal.

Table S2. Complete primary interval-specific GEE estimates

Marker	Interval	n	Difference % (95% CI)	p	q
CRP	0–24 h	179	8.4 (-3.7 to 22.0)	0.184	0.584
CRP	24–48 h	140	5.9 (-5.8 to 19.0)	0.339	0.584
CRP	48–72 h	138	5.5 (-7.9 to 20.9)	0.440	0.626
CRP	72–96 h	123	7.5 (-7.2 to 24.4)	0.334	0.584
D-dimer	0–24 h	88	-12.8 (-26.4 to 3.2)	0.111	0.571
D-dimer	24–48 h	59	-4.5 (-20.2 to 14.4)	0.620	0.769
D-dimer	48–72 h	45	-14.3 (-28.7 to 3.0)	0.100	0.571
D-dimer	72–96 h	47	-14.4 (-29.3 to 3.6)	0.110	0.571
INR	0–24 h	176	-0.6 (-2.2 to 1.0)	0.451	0.626
INR	24–48 h	74	-1.1 (-3.4 to 1.2)	0.338	0.584
INR	48–72 h	82	1.4 (-0.5 to 3.2)	0.143	0.571
INR	72–96 h	60	1.3 (-1.3 to 4.0)	0.340	0.584
IL-6	0–24 h	123	-2.8 (-26.0 to 27.6)	0.838	0.875
IL-6	24–48 h	40	-35.9 (-49.4 to -18.8)	<0.001	0.006
IL-6	48–72 h	40	-10.6 (-33.9 to 21.0)	0.470	0.626
IL-6	72–96 h	42	-11.5 (-35.1 to 20.9)	0.444	0.626
PCT	0–24 h	178	3.7 (-15.1 to 26.6)	0.722	0.788
PCT	24–48 h	133	3.6 (-13.1 to 23.6)	0.690	0.788
PCT	48–72 h	126	8.6 (-7.5 to 27.6)	0.313	0.584
PCT	72–96 h	115	9.7 (-8.3 to 31.2)	0.313	0.584
WBC	0–24 h	185	-0.6 (-7.3 to 6.6)	0.876	0.876
WBC	24–48 h	129	-3.5 (-9.2 to 2.6)	0.260	0.584
WBC	48–72 h	129	1.5 (-4.8 to 8.3)	0.641	0.769
WBC	72–96 h	112	5.1 (-1.4 to 12.1)	0.128	0.571

Estimates are modeled percentage differences per 5 kg/m² higher BMI, adjusted for age, sex, and calendar time. Each patient's arithmetic within-interval mean was $\log_2$-transformed; percentage differences are $100 \times (2^{\beta} - 1)$, where β denotes the relevant interval-specific coefficient or contrast. These are between-patient BMI-associated differences, not within-patient percentage changes over time. All 24 p values form one Benjamini–Hochberg family. The displayed 95% confidence intervals are pointwise and are not multiplicity-adjusted. Patient and observation counts are given in main Table 3. All intervals are left-inclusive and right-exclusive.

Table S3. Mortality sensitivity and interaction estimates

Analysis / term	Patients / deaths	Slopes	OR (95% CI)	p
Unadjusted	201/80	1	1.02 (0.82–1.25)	0.886
Primary: age, sex, calendar time	201/80	4	1.02 (0.82–1.26)	0.868
Severity conditional: primary + APACHE II	201/80	5	1.02 (0.82–1.26)	0.874
Respiratory sensitivity: APACHE + A–a gradient	201/80	6	1.00 (0.80–1.25)	0.990
Respiratory sensitivity: APACHE + P/F ratio	201/80	6	1.01 (0.81–1.25)	0.960
Treatment conditional: severity + steroids/ventilation	201/80	7	1.04 (0.84–1.29)	0.741
Calendar categorical sensitivity	201/80	5	1.04 (0.84–1.29)	0.716
Extended adjustment	199/79	10	0.99 (0.78–1.24)	0.902
BMI 25 to <30 vs <25 kg/m²	201/80	5	1.00 (0.36–2.78)	0.995
BMI ≥30 vs <25 kg/m²	201/80	5	0.97 (0.39–2.43)	0.944
BMI × age interaction (per 10 years)	201/80	5	0.96 (0.81–1.13)	0.586
BMI × sex interaction (female vs male)	201/80	5	0.94 (0.61–1.44)	0.764
BMI × diabetes interaction (present vs absent)	201/80	6	0.90 (0.57–1.42)	0.661

ICU mortality was ascertained for all 201 patients: 80 deaths and 121 survivors. The extended model uses 199 complete cases and 79 deaths; this does not alter the full-cohort outcome count. Interaction ORs are ratios of BMI odds ratios per 10-year age increment, for female versus male sex, or for diabetes presence. They are exploratory and do not establish absence of effect modification. Add one intercept to the slope count for the total number of coefficients. BMI-category contrasts adjust for age, sex, and calendar time and use BMI <25 kg/m² as the reference. Full coefficients are available in the statistical workbook.

Table S4. Observation availability and longitudinal profiles

Marker	Interval	Observed / 201	Exited before interval	Missing while in ICU	<25	25 to <30	≥30
CRP	0–24 h	179/201	0	22	20/23 (87.0%)	47/53 (88.7%)	112/125 (89.6%)
CRP	24–48 h	140/201	9	52	16/23 (69.6%)	32/53 (60.4%)	92/125 (73.6%)
CRP	48–72 h	138/201	13	50	12/23 (52.2%)	35/53 (66.0%)	91/125 (72.8%)
CRP	72–96 h	123/201	14	64	14/23 (60.9%)	35/53 (66.0%)	74/125 (59.2%)
D-dimer	0–24 h	88/201	0	113	11/23 (47.8%)	20/53 (37.7%)	57/125 (45.6%)
D-dimer	24–48 h	59/201	9	133	6/23 (26.1%)	15/53 (28.3%)	38/125 (30.4%)
D-dimer	48–72 h	45/201	13	143	7/23 (30.4%)	13/53 (24.5%)	25/125 (20.0%)
D-dimer	72–96 h	47/201	14	140	4/23 (17.4%)	13/53 (24.5%)	30/125 (24.0%)
INR	0–24 h	176/201	0	25	19/23 (82.6%)	46/53 (86.8%)	111/125 (88.8%)
INR	24–48 h	74/201	9	118	11/23 (47.8%)	19/53 (35.8%)	44/125 (35.2%)
INR	48–72 h	82/201	13	106	7/23 (30.4%)	16/53 (30.2%)	59/125 (47.2%)
INR	72–96 h	60/201	14	127	9/23 (39.1%)	13/53 (24.5%)	38/125 (30.4%)
IL-6	0–24 h	123/201	0	78	15/23 (65.2%)	28/53 (52.8%)	80/125 (64.0%)
IL-6	24–48 h	40/201	9	152	4/23 (17.4%)	9/53 (17.0%)	27/125 (21.6%)
IL-6	48–72 h	40/201	13	148	4/23 (17.4%)	13/53 (24.5%)	23/125 (18.4%)
IL-6	72–96 h	42/201	14	145	4/23 (17.4%)	13/53 (24.5%)	25/125 (20.0%)
PCT	0–24 h	178/201	0	23	19/23 (82.6%)	46/53 (86.8%)	113/125 (90.4%)
PCT	24–48 h	133/201	9	59	14/23 (60.9%)	30/53 (56.6%)	89/125 (71.2%)
PCT	48–72 h	126/201	13	62	12/23 (52.2%)	33/53 (62.3%)	81/125 (64.8%)
PCT	72–96 h	115/201	14	72	14/23 (60.9%)	33/53 (62.3%)	68/125 (54.4%)
WBC	0–24 h	185/201	0	16	20/23 (87.0%)	48/53 (90.6%)	117/125 (93.6%)
WBC	24–48 h	129/201	9	63	16/23 (69.6%)	30/53 (56.6%)	83/125 (66.4%)
WBC	48–72 h	129/201	13	59	13/23 (56.5%)	30/53 (56.6%)	86/125 (68.8%)
WBC	72–96 h	112/201	14	75	14/23 (60.9%)	32/53 (60.4%)	66/125 (52.8%)

Observed counts denote patients with an exact positive interval summary. ICU exits are defined by recorded discharge times before interval start. Censored-only intervals do not contribute exact positive summaries. Intervals are [0,24), [24,48), [48,72), and [72,96) hours after admission. The profile table below reports how many of the 201 patients contributed zero through four eligible intervals for each biomarker.

Marker	0	1	2	3	4	Obs. / patients
CRP	5	17	35	83	61	580/196
D-dimer	74	52	47	19	9	239/127
INR	7	61	79	43	11	392/194
IL-6	52	85	39	18	7	245/149
PCT	6	20	42	84	49	552/195
WBC	5	21	47	72	56	555/196

Table S5. Characteristics of patients with and without an observed IL-6 value

Interval	Characteristic	Measured	Without an observed value, including prior ICU exits	|SMD|
0–24 h	Age, years	67.0 [60.0–73.0] (n=123)	66.5 [60.0–74.8] (n=78)	0.176
0–24 h	BMI, kg/m²	31.0 [28.6–35.2] (n=123)	30.8 [26.4–34.2] (n=78)	0.072
0–24 h	APACHE II score	30.0 [25.0–34.0] (n=123)	31.0 [20.0–36.0] (n=78)	0.011
0–24 h	PaO_2/FiO_2, mmHg	108.0 [75.4–163.0] (n=123)	98.9 [68.2–147.6] (n=78)	0.129
0–24 h	Female sex	54/123 (43.9%)	33/78 (42.3%)	0.032
0–24 h	Corticosteroids, any time	111/123 (90.2%)	66/78 (84.6%)	0.170
0–24 h	Mechanical ventilation, any time	113/123 (91.9%)	70/78 (89.7%)	0.074
0–24 h	ICU death	47/123 (38.2%)	33/78 (42.3%)	0.084
24–48 h	Age, years	66.0 [56.0–70.0] (n=40)	67.0 [60.0–74.0] (n=161)	0.238
24–48 h	BMI, kg/m²	31.4 [28.9–34.1] (n=40)	30.9 [27.0–35.0] (n=161)	0.120
24–48 h	APACHE II score	29.5 [20.8–32.5] (n=40)	31.0 [22.0–35.0] (n=161)	0.257
24–48 h	PaO_2/FiO_2, mmHg	106.0 [76.4–130.0] (n=40)	101.0 [71.0–163.1] (n=161)	0.220
24–48 h	Female sex	21/40 (52.5%)	66/161 (41.0%)	0.232
24–48 h	Corticosteroids, any time	38/40 (95.0%)	139/161 (86.3%)	0.301
24–48 h	Mechanical ventilation, any time	37/40 (92.5%)	146/161 (90.7%)	0.066
24–48 h	ICU death	15/40 (37.5%)	65/161 (40.4%)	0.059
48–72 h	Age, years	65.5 [61.5–70.0] (n=40)	68.0 [60.0–74.0] (n=161)	0.087
48–72 h	BMI, kg/m²	30.8 [27.9–34.5] (n=40)	31.0 [27.3–35.0] (n=161)	0.015
48–72 h	APACHE II score	27.0 [19.0–32.0] (n=40)	31.0 [25.0–35.0] (n=161)	0.446
48–72 h	PaO_2/FiO_2, mmHg	108.8 [75.2–159.5] (n=40)	101.0 [71.7–157.8] (n=161)	0.035
48–72 h	Female sex	15/40 (37.5%)	72/161 (44.7%)	0.147
48–72 h	Corticosteroids, any time	38/40 (95.0%)	139/161 (86.3%)	0.301
48–72 h	Mechanical ventilation, any time	35/40 (87.5%)	148/161 (91.9%)	0.146
48–72 h	ICU death	12/40 (30.0%)	68/161 (42.2%)	0.257
72–96 h	Age, years	66.5 [62.0–70.8] (n=42)	67.0 [60.0–73.5] (n=159)	0.029
72–96 h	BMI, kg/m²	30.0 [27.2–32.1] (n=42)	31.0 [27.5–36.0] (n=159)	0.198
72–96 h	APACHE II score	27.0 [19.5–32.0] (n=42)	31.0 [25.0–35.0] (n=159)	0.398
72–96 h	PaO_2/FiO_2, mmHg	93.7 [71.9–130.1] (n=42)	104.0 [74.4–163.0] (n=159)	0.206
72–96 h	Female sex	18/42 (42.9%)	69/159 (43.4%)	0.011
72–96 h	Corticosteroids, any time	38/42 (90.5%)	139/159 (87.4%)	0.098
72–96 h	Mechanical ventilation, any time	35/42 (83.3%)	148/159 (93.1%)	0.306
72–96 h	ICU death	19/42 (45.2%)	61/159 (38.4%)	0.140

Values are median [IQR] (n) or n/N (%). The comparator comprises patients without an observed value, including prior ICU exits; it is not restricted to patients still eligible for observation. Table S4 distinguishes prior exits from absent measurement during ICU stay. The Measured_vs_unmeasured sheet of Final_Statistics.xlsx reports all biomarkers and intervals. Mortality refers to the clinically reviewed index ICU outcome. Treatment indicators describe any recorded exposure during the stay, not necessarily exposure before the assay.

Table S6A. IL-6 24–48-hour sensitivity estimates

Sensitivity	Obs. / patients	n in interval	Difference % (95% CI)	p	q
Unadjusted	245/149	40	-36.0 (-49.6 to -18.6)	<0.001	0.006
Severity conditional	245/149	40	-35.7 (-49.5 to -18.1)	<0.001	0.008
Treatment conditional	245/149	40	-35.7 (-49.7 to -17.7)	<0.001	0.011
Independence working correlation	245/149	40	-49.9 (-60.9 to -35.7)	<0.001	<0.001
Bias-reduced sandwich	245/149	40	-35.9 (-50.5 to -17.1)	<0.001	0.017
Categorical calendar	245/149	40	-35.9 (-49.7 to -18.4)	<0.001	0.008
Inverse probability of observation weighting	245/149	40	-48.4 (-65.2 to -23.7)	<0.001	0.011
IL-6 at least two measurements	160/64	34	-43.1 (-55.6 to -27.0)	<0.001	—
Exclude documented tocilizumab (other exposure unknown)	225/137	37	-38.1 (-51.0 to -21.9)	<0.001	—
Censoring bound substitution	253/151	42	-37.2 (-50.7 to -19.9)	<0.001	0.004

All estimates are modeled percentage differences per 5 kg/m² higher BMI. Each complete six-biomarker sensitivity analysis has a separate false-discovery-rate family of 24 interval contrasts; IL-6-only restrictions are exploratory and have unadjusted p values without q values. The displayed 95% confidence intervals are pointwise and are not multiplicity-adjusted. Boundary substitution assigns the reported limit to a censored value (<L or ≤L → L; >U or ≥U → U), combines it with exact results in the arithmetic patient–biomarker–interval mean, and then $\log_2$-transforms positive means. This assumption does not recover true censored concentrations. Seven complete IL-6 profiles were insufficient for an 11-coefficient model. Five actual-gap AR(1) fits failed; that incomplete family is not interpreted.

Table S6B. Joint BMI tests under alternative working correlations

Biomarker	Patients / observations	Exchangeable p	Exchangeable q	Independence p	Independence q
CRP	196 / 580	0.641	0.651	0.779	0.814
D-dimer	127 / 239	0.227	0.384	0.0017	0.0051
INR	194 / 392	0.256	0.384	0.772	0.814
IL-6	149 / 245	0.0001	0.0007	<0.0001	<0.0001
PCT	195 / 552	0.651	0.651	0.814	0.814
WBC	196 / 555	0.0006	0.0017	0.010	0.020

Both Gaussian GEE specifications adjust for age, sex, and calendar time and include BMI-by-interval interaction terms. Each joint BMI test has four degrees of freedom. Benjamini–Hochberg adjustment is performed separately across the six biomarkers within each correlation specification; these q values are not the 24-contrast interval-family q values. The D-dimer comparison uses the same 127 patients and 239 observations in both models. Complete-precision values and the reproducible summary script accompany the statistical archive.

Table S7. Inverse-probability-of-observation diagnostics

Marker	Interval	Eligible / observed	P(observed) range	Weight caps	Effective n
CRP	0–24 h	201/179	0.407–0.998	0.895–1.554	176.3
CRP	24–48 h	192/140	0.383–0.920	0.802–1.487	137.0
CRP	48–72 h	188/138	0.535–0.864	0.870–1.233	136.7
CRP	72–96 h	187/123	0.420–0.825	0.809–1.480	121.4
D-dimer	0–24 h	201/88	0.029–0.935	0.472–2.172	74.6
D-dimer	24–48 h	192/59	0.042–0.880	0.395–3.703	38.7
D-dimer	48–72 h	188/45	0.003–0.765	0.320–2.557	33.8
D-dimer	72–96 h	187/47	0.043–0.616	0.461–2.134	40.0
INR	0–24 h	201/176	0.266–0.999	0.878–1.656	172.5
INR	24–48 h	192/74	0.230–0.641	0.639–1.506	70.5
INR	48–72 h	188/82	0.270–0.667	0.690–1.411	79.8
INR	72–96 h	187/60	0.076–0.728	0.464–1.965	52.9
IL-6	0–24 h	201/123	0.105–0.967	0.646–2.646	103.9
IL-6	24–48 h	192/40	0.010–0.867	0.261–2.055	31.0
IL-6	48–72 h	188/40	0.030–0.590	0.387–2.429	32.2
IL-6	72–96 h	187/42	0.008–0.614	0.378–3.594	28.9
PCT	0–24 h	201/178	0.370–0.998	0.890–1.541	175.1
PCT	24–48 h	192/133	0.298–0.981	0.721–1.696	126.3
PCT	48–72 h	188/126	0.321–0.902	0.760–1.721	122.1
PCT	72–96 h	187/115	0.296–0.751	0.826–1.429	113.2
WBC	0–24 h	201/185	0.489–0.998	0.924–1.351	183.9
WBC	24–48 h	192/129	0.406–0.869	0.775–1.428	126.3
WBC	48–72 h	188/129	0.312–0.936	0.760–1.543	126.5
WBC	72–96 h	187/112	0.205–0.898	0.693–2.018	104.9

Eligible patients remained in the ICU at interval start. Observation models include BMI, age, sex, APACHE II, and calendar time; follow-up models additionally include baseline $\log_2$ biomarker concentration and its missingness indicator. Median replacement applies only to a missing baseline predictor in this auxiliary model. Stabilized weights equal the marginal observed fraction divided by the fitted observation probability and are capped at the 1st and 99th percentiles among observed patients. Independence GEE uses robust covariance conditional on the estimated weights; it does not propagate all observation-model uncertainty. Effective n = (sum of weights)^2 / sum of squared weights. Intervals are left-inclusive and right-exclusive.

Table S8. Calendar-period description

Period	N	BMI	ICU deaths n (%)	Steroids n/N (%)	IL-6 first-block n
2020 Sep–Dec	81	31.0 [27.7–36.0] (n=81)	31 (38.3)	70/81 (86.4%)	26
2021 Jan–Mar	87	30.1 [27.0–33.1] (n=87)	38 (43.7)	77/87 (88.5%)	72
2021 Apr–Jun	33	31.2 [30.0–36.0] (n=33)	11 (33.3)	30/33 (90.9%)	25

The historical screening frame was 3 March 2020–1 July 2021. Actual included ICU admission dates extended from 25 September 2020 to 19 June 2021, consistent with the unit beginning to admit patients with critical COVID-19 in September 2020. Calendar time in the primary model is elapsed admission time divided by 91.3125 days. Period sensitivity uses September–December 2020, January–March 2021, and April–June 2021. These are calendar bands, not inferred viral variants. The itemized original screening log was not available for independent reconstruction of exclusion reasons.

Table S9. Additional clinical documentation and TISS

Variable	Positive n	Coverage	Interpretation
Tocilizumab	18	196 screened; 201 cohort	Unmentioned status unknown
Remdesivir	17	196 screened; 201 cohort	Unmentioned status unknown
Baricitinib	0	196 screened; 201 cohort	Unmentioned status unknown
COVID-19 vaccination	5	196 screened; 201 cohort	Unmentioned status unknown
Chronic kidney disease	22	196 screened; 201 cohort	Unmentioned status unknown
Corticosteroid documentation	51	196 screened; 201 cohort	Unmentioned status unknown
Anticoagulant documentation	35	196 screened; 201 cohort	Unmentioned status unknown
TISS mechanical ventilation	180	196	Whole stay
TISS single vasoactive drug	167	196	Whole stay
TISS multiple vasoactive drugs	128	196	Whole stay
TISS either vasoactive category	183	196	Whole stay
TISS renal replacement therapy	36	196	Whole stay

Counts represent positive documentation, not complete exposure prevalence. The narrative/TISS export covers 196 of the 201 cohort patients; this differs from the all-patient review performed for ICU outcome ascertainment. Chronic kidney disease combines explicit narrative documentation and usable N18 code lists. TISS comprises 2,764 entries, with median 11.5 [7.75–17] entries per patient (range, 1–64). Complete day-specific treatment timing was unavailable. Undocumented exposure remains unknown and is not classified as absent.

Table S10. Bootstrap and spline diagnostics

Model	Successful resamples	BMI OR interval	Apparent AUC	Corrected AUC	Corrected slope
Primary adjustment	1000	0.81–1.26	0.557	0.493	0.305
Severity adjustment	1000	0.78–1.25	0.556	0.480	0.264

There were 1,000 patient-level bootstrap resamples; random seed 4529624. The empirical BMI interval comprises the 2.5th–97.5th percentiles of bootstrap ORs. The AUC correction subtracts mean bootstrap training-minus-test optimism. The reported corrected calibration slope is the mean slope when bootstrap model predictors are evaluated in the original sample. All models use the final 80-death/121-survivor ICU outcome classification. These diagnostics do not establish external predictive validity or absence of bias. The restricted cubic spline has knots at BMI 24.442, 31.000, and 39.840 kg/m², one nonlinear degree of freedom, likelihood-ratio statistic 1.022, and $p = 0.312$. It estimates two BMI coefficients plus age, sex, calendar time, and an intercept. Figure S2 uses BMI 30 kg/m² as the reference.

Table S11. Clinical cohort, source linkage, and analytical coverage

Source	Cohort identifiers with data	Successfully linked patients	Source records / review	Usable analytical information
Main study registry	201/201	201	201 patient-level records	All 201 patients contribute to primary mortality analysis; model-specific complete cases are reported separately.
Hospital report and clinical outcome review	201/201	201 reviewed patients	Patient-level outcome report and discharge summaries/medical documentation reviewed for all 201 patients	80 ICU deaths and 121 ICU survivors. This review does not imply complete biomarker or TISS exports.
Structured laboratory export	198/201	198	102,153 laboratory records before biomarker/time/result restrictions	2,563 eligible patient–biomarker–interval summaries: CRP 580/196; D-dimer 239/127; INR 392/194; IL-6 245/149; PCT 552/195; WBC 555/196 (observations/patients).
Narrative export	196/201	196	196 patient-linked narrative records	Positive documentation counts for diagnoses, medications, and vaccination; undocumented status remains unknown.
TISS export	196/201	196	2,764 TISS entries summarized in patient-level profiles	196 whole-stay profiles; reliable baseline or pre-assay treatment timing was unavailable.

The cohort denominator remains 201. Identifiers with source records and successfully linked patients are separate concepts; the reported counts coincide for the laboratory, narrative, and TISS extracts. The available summary does not separately count every failed linkage attempt or establish why individual source records were absent from an export. Clinical documentation review for outcome ascertainment is a different process from structured-data export. A linked patient may contribute no eligible value for a specific biomarker or interval. The laboratory patient counts are marker-specific and must not be summed across biomarkers.

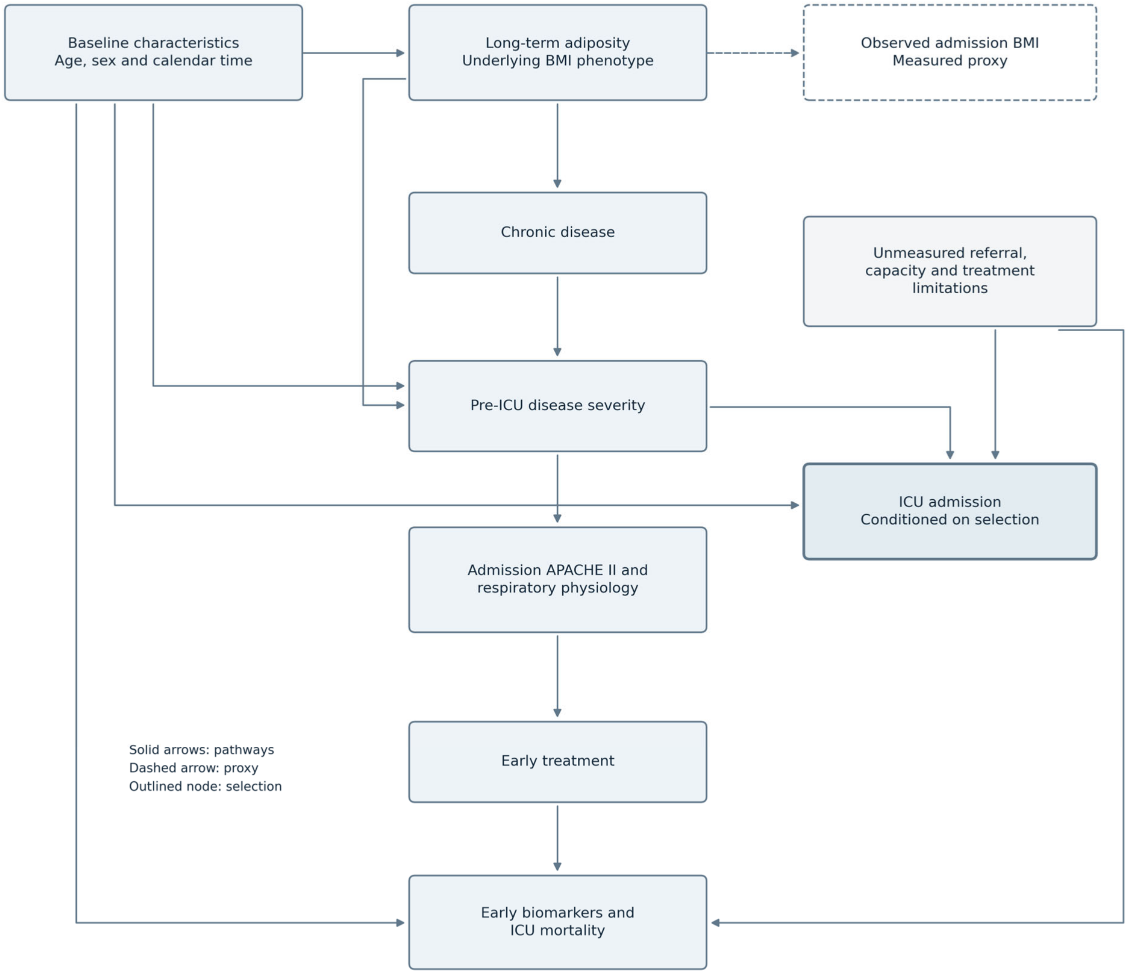

Figure S1. Conceptual relationships and ICU selection. Admission BMI is a proxy for antecedent adiposity. Arrows describe hypothesized relationships, not effects estimated from this study. Conditioning on ICU admission may induce bias. Age/sex/calendar adjustment does not identify a population causal effect.

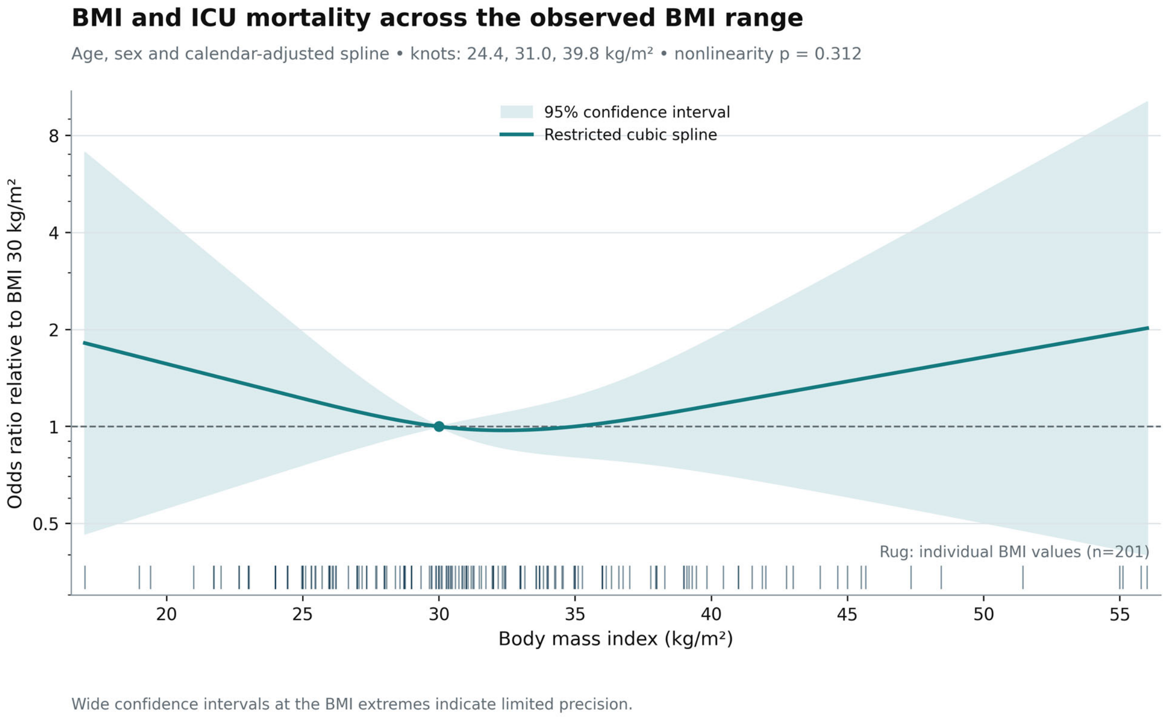

Figure S2. Exploratory BMI spline with pointwise 95% confidence band and the observed exposure distribution. The odds-ratio reference is BMI 30 kg/m². Sparse observations at the distributional extremes limit precision; a nonsignificant nonlinearity test does not establish a linear association.

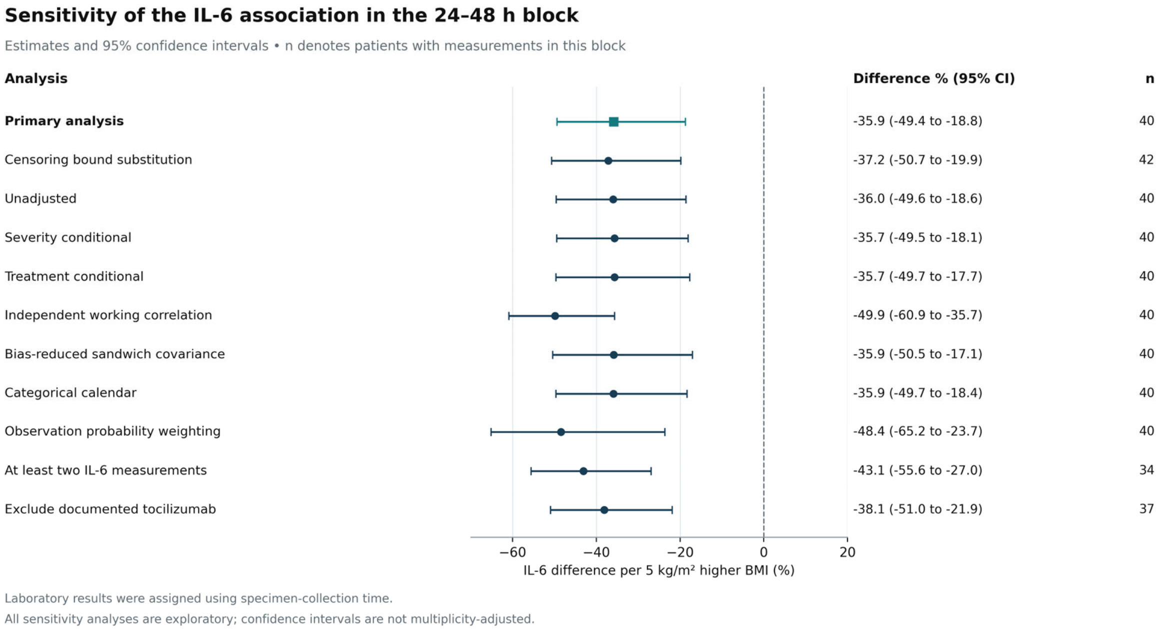

Figure S3. Sensitivity estimates for the IL-6 24–48-hour interval. All rows represent modeled percentage differences per 5 kg/m² higher BMI. The displayed 95% confidence intervals are pointwise and are not multiplicity-adjusted. Specimen-collection time defines the interval [24,48) hours after ICU admission. In the boundary-substitution analysis, reported lower and upper limits replace censored values before arithmetic interval averaging and $\log_2$ transformation; substituted values are not exact measurements.
